# Recurrent Pneumothorax With Extensive Subcutaneous Emphysema During the Perioperative Course of Type A Aortic Dissection: A Case Report

**DOI:** 10.1002/ccr3.72468

**Published:** 2026-04-07

**Authors:** Qiulin Ran, Zanxin Wang, Minxin Wei, Bin You

**Affiliations:** ^1^ Department of Cardiovascular Surgery Beijing Anzhen Hospital, Capital Medical University, Beijing Institute of Heart Lung and Blood Vessel Diseases Beijing China; ^2^ The University of Hong Kong‐Shenzhen Hospital Shenzhen China

**Keywords:** ascending aorta, catheterization, central venous, dissection, pneumothorax, sternotomy, subcutaneous emphysema, thoracostomy

## Abstract

Acute type A aortic dissection (AoD) requires urgent surgical treatment, and perioperative complications may result from both the underlying disease and procedure‐related factors. Here, we report a 29‐year‐old man with acute type A AoD who developed right pneumothorax before undergoing emergency aortic repair via median sternotomy. Postoperatively, the pneumothorax recurred and rapidly progressed to extensive cervical, thoracic, and abdominal subcutaneous emphysema (SE). Recurrent pneumothorax persisted despite chest drainage. Subsequent evaluation revealed right apical pulmonary bullae; however, the overall clinical course suggested a multifactorial etiology, including persistent coughing, positive‐pressure ventilation, internal jugular venous catheterization, wound air leakage, and drainage‐related factors. Mediastinal pleural disruption, along with a low body mass index (BMI) and limited soft‐tissue buffering, may have further facilitated air dissection along tissue planes and increased the severity of SE. After 2 weeks of chest tube drainage, the SE gradually resolved, and no further pneumothorax was observed after tube removal. This case emphasizes the importance of prompt recognition, repeated imaging assessment, and individualized drainage management in patients with perioperative pneumothorax and extensive SE undergoing cardiac surgery.

## Key Clinical Message

Perioperative pneumothorax with extensive subcutaneous emphysema during surgery for acute type A aortic dissection may be multifactorial. Careful assessments of preexisting lung abnormalities, perioperative triggers, and drainage‐related factors are essential for timely recognition, appropriate management, and prevention of further deterioration.

**Abbreviations**
AoD: aortic dissection
BMI: body mass index
ICU: intensive care unit
SE: subcutaneous emphysema

## 1 Introduction

Acute type A aortic dissection (AoD) is a surgical emergency associated with high mortality if not promptly treated [1]. Emergency surgical repair remains the standard treatment, although perioperative complications are common because of the severity of the underlying disease and the complexity of the procedure. These complications typically include bleeding, organ malperfusion, neurological injury, acute kidney injury, and infection [2, 3]. In contrast, perioperative pneumothorax with extensive subcutaneous emphysema (SE) is less commonly observed and may result in diagnostic and management challenges. SE has been described in a wide range of settings, including respiratory disorders, trauma, vomiting, labor, and thoracic or airway‐related invasive procedures [4, 5, 6]. In patients undergoing cardiac surgery, pneumothorax and SE may be multifactorial rather than solely procedure‐related, particularly in the presence of preexisting pulmonary abnormalities, perioperative triggers, or drainage‐related factors.

Here, we report the case of a man with acute type A AoD who developed pneumothorax before emergency aortic repair via median sternotomy. During the postoperative course, the pneumothorax recurred and was followed by rapidly progressive SE involving the neck, chest, and abdominal wall. Recurrent pneumothorax persisted despite chest drainage. This case highlights the importance of prompt recognition, careful differential assessment, repeated imaging evaluation, and individualized drainage management for perioperative pneumothorax and SE in complex cardiac surgical patients.

## 2 Case History/Examination

**FIGURE 1:**
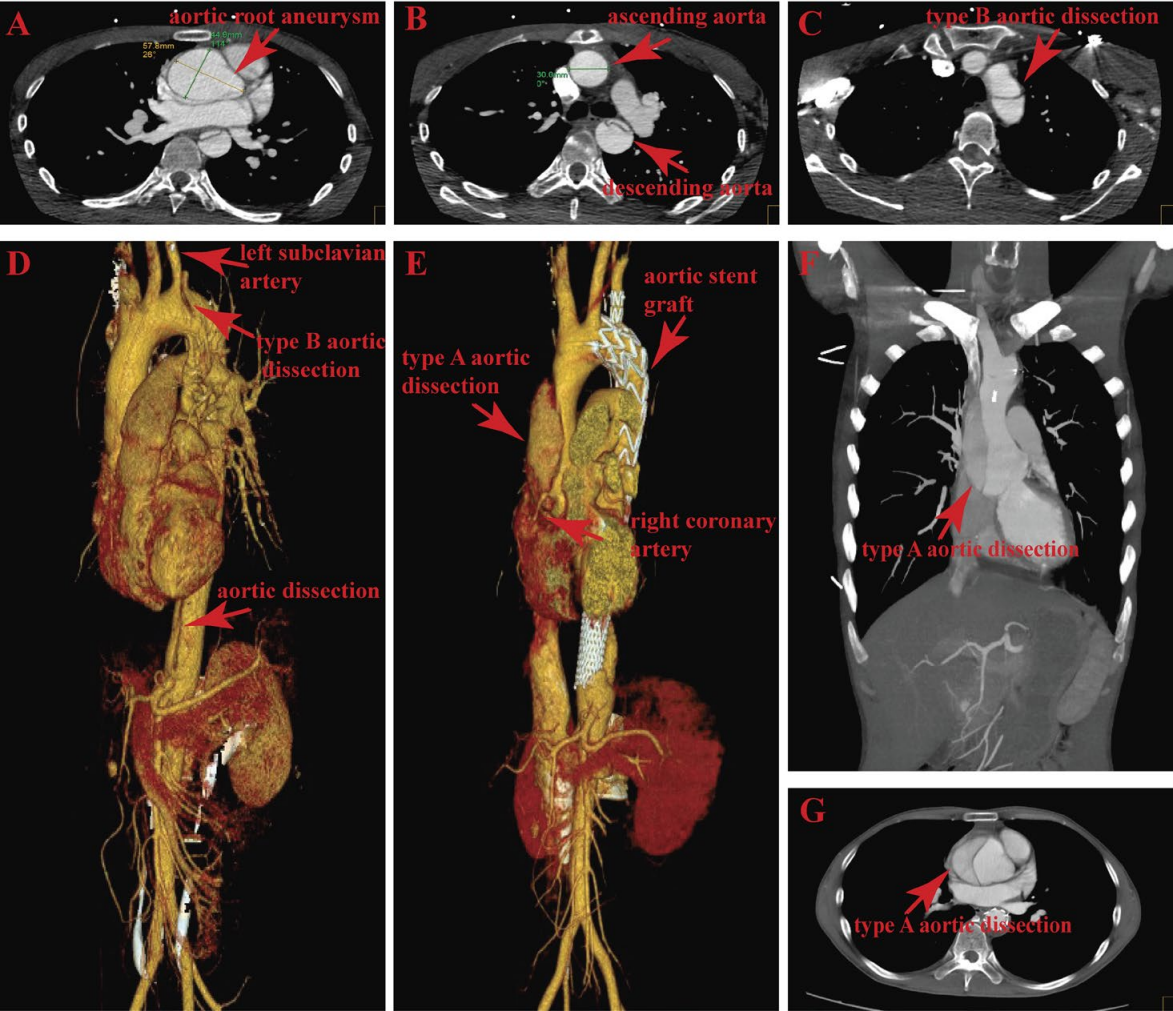
Computed tomography angiography images. (A, B) The maximum diameter of the aortic root aneurysm was 57.8 mm (arrow). (C, D) Imaging of type B aortic dissection (arrow). (E–G) Imaging of type A aortic dissection (arrow).

A 29‐year‐old man presented to our emergency department with persistent chest pain and chest tightness. Additionally, he had a 2‐day history of cough before admission. He had no history of smoking. On admission, he weighed 52.5 kg and was 180 cm tall, which corresponded to a low body mass index (BMI) of 16.2 kg/m^2^. His vital signs were as follows: temperature, 37.1°C; heart rate, 74 beats/min; blood pressure, 149/64 mmHg; and respiratory rate, 20 breaths/min. Before admission to the intensive care unit (ICU), urgent computed tomography angiography was performed and revealed acute type A AoD, with possible involvement of the origin of the right coronary artery (Figure 1F,G).

Three weeks earlier, he had been diagnosed at our institution with an aortic root aneurysm (Figure 1A,B) and acute type B AoD (Figure 1B–D), for which he underwent emergency thoracic endovascular stent‐graft repair using a Castor stent graft (C302412‐2002510) (Figure 1E). After stent implantation, his postoperative course was complicated by poor adherence to medical treatment and intermittent low‐grade fever. Due to these issues, he was considered to be unsuitable for immediate surgical treatment of the aortic root aneurysm and was discharged with a plan for interval recovery before reassessment. The interval between his previous discharge and the current admission was 10 days.

## 3 Differential Diagnosis, Investigations and Treatment

During preoperative preparation in the ICU, the patient experienced persistent coughing. To reduce the risk of cough‐related hemodynamic deterioration and possible aortic rupture, endotracheal intubation and positive‐pressure ventilation were initiated. Central venous access was then attempted via the right internal jugular vein under ultrasound guidance. During aspiration, air was noted in the catheter, thus eliciting concern for pleural injury. A bedside chest radiograph subsequently demonstrated a right pneumothorax with approximately 40% lung compression, thereby representing the first documented occurrence of pneumothorax during this hospitalization. A chest tube was inserted through the fifth intercostal space at the right midaxillary line.

**FIGURE 2:**
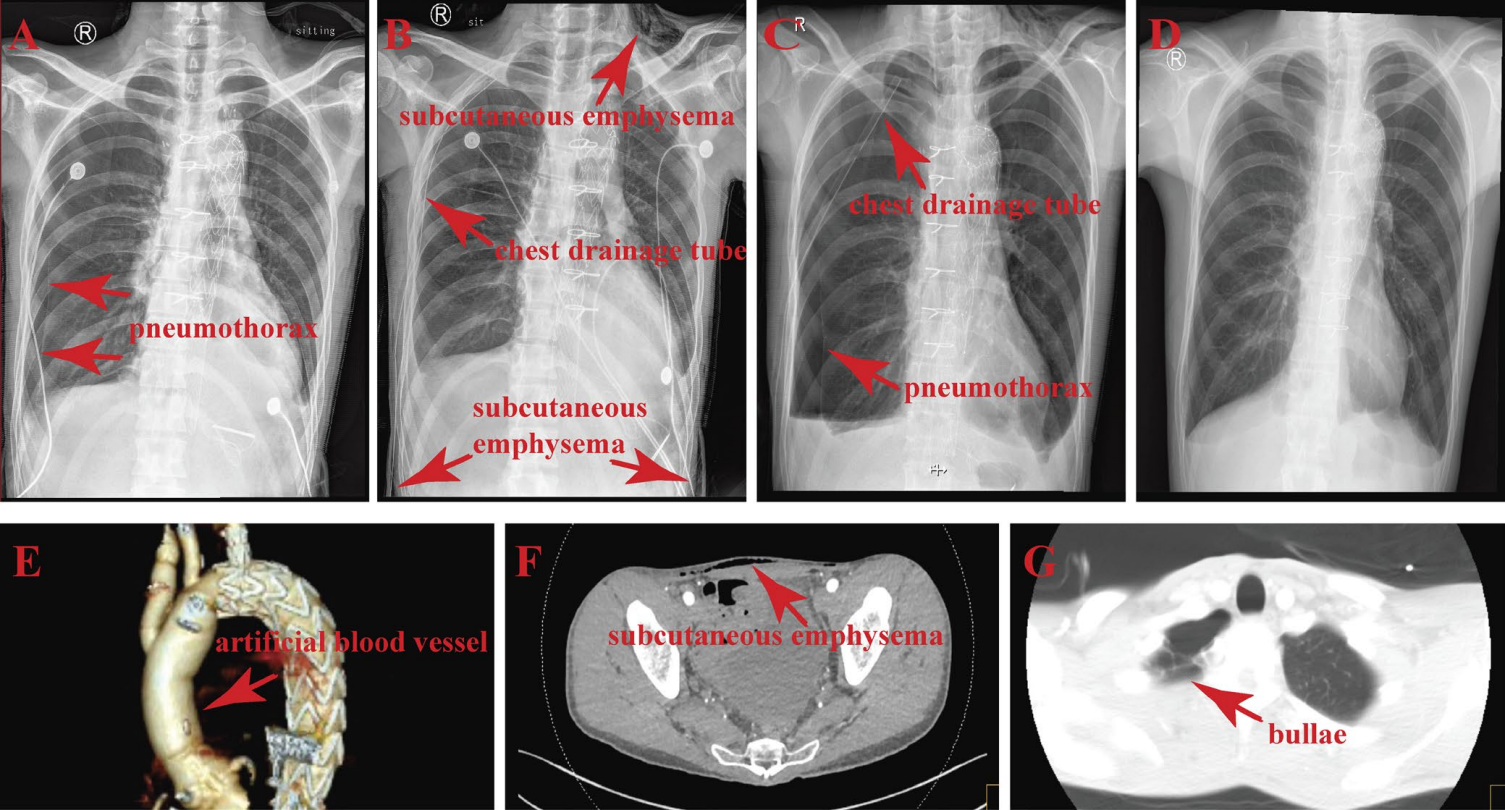
Computed tomography angiography images and chest radiographs. (A–C, F) Imaging of pneumothorax and cervical and abdominal SE (arrow). (D) SE had been almost completely absorbed at the time of discharge. (E) Three‐dimensional reconstruction of the ascending aortic graft (arrow). (G) Right apical pulmonary bullae (arrow).

The patient subsequently underwent emergency valve‐sparing aortic root replacement, coronary artery bypass grafting, and total arch replacement with a prosthetic vascular graft (Figure 2E). Due to the fact that the right mediastinal pleura had been widely opened intraoperatively, the chest tube that had been placed for the pneumothorax was removed. Three drainage tubes were then placed below the xiphoid process for the pericardial space, mediastinum, and right pleural cavity.

On postoperative day five, the daily pleural drainage volume was determined to be less than 100 mL, and no air leakage was observed in the water‐seal chamber. Therefore, the chest tubes were removed. The patient had very little subcutaneous tissue, and the drainage site appeared to be poorly sealed after tube removal. The wound was resutured and covered with petrolatum gauze. Soon afterward, he developed a sudden episode of severe coughing, accompanied by audible air leakage from the wound. Rapidly progressive SE was then noted over the neck, chest, and abdominal wall (Figure 2B,F). The patient complained of marked neck and abdominal discomfort as well as dysphagia. Bedside chest radiography confirmed recurrent right pneumothorax (Figure 2A), thus representing the second documented case of pneumothorax during hospitalization. A chest tube was reinserted through the previous incision at the right midaxillary line (Figure 2B), after which the degree of cervical discomfort slightly improved.

One day after reinsertion, no air leakage was observed in the water‐seal chamber. The chest tube was clamped for 24 h, and the patient remained asymptomatic. Before planned tube removal, repeat chest radiography was performed to confirm the resolution of the pneumothorax. However, recurrent pneumothorax was again identified, thus representing the third documented episode during this hospitalization (Figure 2C). A thoracic surgery consultation was obtained; review of the lung‐window images from computed tomography angiography demonstrated right upper lobe apical bullae (Figure 2G), and surgical management was discussed as an alternative option in the event of further clinically significant recurrence. Given the clinical course and imaging findings, the recurrent pneumothorax was considered to be likely multifactorial in nature, with contributions from preexisting apical bullae, persistent coughing, positive‐pressure ventilation, perioperative pleural disruption, wound air leakage, and drainage‐related factors.

## 4 Conclusion and Results (Outcome and Follow‐Up)

After 12 days of pleural drainage, the chest tube was clamped for 48 h, and no recurrent pneumothorax was observed during the clamping period. The tube was subsequently removed, and no immediate recurrence occurred. At that time, most of the SE had been absorbed (Figure 2D). An underlying connective tissue disorder was clinically considered, although confirmatory genetic testing was not performed. At 3 months after discharge, the SE had completely resolved, and there was no evidence of recurrent pneumothorax.

This case highlights the fact that perioperative pneumothorax during emergency surgery for acute type A AoD may arise from multiple interacting factors rather than a single identifiable cause. Persistent coughing, mechanical ventilation, central venous catheterization, preexisting bullous change, wound air leakage, and drainage‐related factors should all be considered. Patients with a low BMI, mediastinal pleural disruption, and limited soft‐tissue buffering may be particularly vulnerable to extensive SE once a pneumothorax develops. Therefore, careful differential assessment, repeated imaging assessment, and individualized drainage management are critical in this setting.

## 5 Discussion

Iatrogenic pneumothorax is a recognized complication in the ICU and might arise after invasive procedures such as central venous catheterization, thoracentesis, pleural biopsy, tracheostomy, bronchoscopy, and positive‐pressure ventilation [7]. The internal jugular vein is commonly preferred for central venous access because it is associated with lower risks of pneumothorax and bleeding than other access sites, and ultrasound guidance can further reduce these risks [8]. However, the risk of pneumothorax remains higher in patients with preexisting pulmonary abnormalities and in those requiring positive‐pressure ventilation [9]. Pulmonary abnormalities associated with connective tissue disorders may include emphysema, bullous change, and spontaneous pneumothorax. Moreover, apical bullae are also frequently observed in tall, thin young adults with primary spontaneous pneumothorax and are not specific to Marfan syndrome [10, 11]. In our patient, the combination of a tall, thin bodily type, with elongated limbs and fingers, apical bullae, aortic root aneurysm, and AoD elicited concern about an underlying connective tissue disorder. Therefore, Marfan syndrome was considered to be clinically relevant, although it could not be confirmed because genetic testing was not performed. In the acute setting, such testing was not expected to alter the emergency surgical strategy, and further genetic evaluation was not pursued by the patient and his family.

In this patient, the cause of the recurrent pneumothorax was considered to be likely multifactorial in nature. Persistent coughing, positive‐pressure ventilation, internal jugular venous catheterization, chest tube removal, possible air leakage from the drainage site, and preexisting apical bullae may have collectively contributed to this event. On readmission, testing for influenza and coronavirus disease 2019 was negative. Although approximately 4 months had elapsed since the coronavirus disease 2019 outbreak at the time of hospitalization, a possible association between the patient's cough and prior coronavirus infection cannot be excluded, as post‐infectious airway vulnerability may have increased his susceptibility to cough [12]. Although the cause of the cough remained unclear, coughing itself is a recognized trigger for pneumothorax and SE; moreover, it may also occur as a nonspecific symptom of pneumothorax [4, 13]. The first case of pneumothorax was suspected to be related to internal jugular venous catheterization because air was noted in the catheter during aspiration; however, this could not be confirmed. As no air leakage was observed and radiography suggested resolution of the pneumothorax, the tube was removed without a prolonged clamping trial. The subsequent recurrence suggests that a clamping trial with close radiographic follow‐up should be considered in similar high‐risk patients. However, recurrent pneumothorax may also occur after chest tube removal, particularly in the presence of intrathoracic pressure fluctuations [14]. In our patient, incomplete sealing of the drainage site and a minimal amount of subcutaneous tissue may have served as a primary source of air leakage and/or acted synergistically with other factors to exacerbate the pneumothorax and SE. In addition, apical bullae were identified in the right upper lobe, although the presence and timing of bullous rupture could not be determined. Overall, the clinical course suggested that the pneumothorax was not attributable to a single factor.

Postoperative SE has been reported after thoracoscopic lung, esophageal, and cardiac procedures; moreover, it is usually mild and conservatively managed [15, 16]. However, in some patients, SE may become extensive, particularly in the presence of pneumothorax or inadequate air drainage. Reported risk factors include a low BMI, low body weight, advanced age, a prolonged operative duration, and factors related to surgical access and lung manipulation [6, 15, 17]. In our patient, markedly reduced subcutaneous and mediastinal adipose tissue may have promoted the dissection of air along fascial planes and contributed to the rapid spread of SE once pneumothorax recurred. This anatomical feature may also have reduced tissue resistance during catheter placement, although its precise contribution cannot be retrospectively determined. Taken together, these observations suggest that mediastinal pleural disruption, along with a low BMI and limited soft‐tissue buffering, may have increased the severity of SE in this case rather than serving as an isolated explanation for its occurrence.

Compared with minimally invasive cardiac surgery, median sternotomy is generally associated with increased postoperative pleural effusion and a higher rate of wound‐related complications [18]. SE after median sternotomy is an uncommon event but has been previously reported. Existing reports suggest that it may develop through different mechanisms, including dissection of alveolar air along bronchovascular and mediastinal tissue planes even in the absence of pneumothorax, as well as postoperative factors such as wound infection or endomyocardial biopsy [19, 20]. These observations suggest that postoperative SE in cardiac surgical patients may result from different underlying mechanisms. However, in our patient, the clinical course was characterized by recurrent pneumothorax and rapidly progressive SE, thus suggesting a mechanism that is distinct from that in patients without pleural air leakage. In conjunction with the other perioperative findings, this comparison further supports a multifactorial explanation rather than a single definitive cause.

Severe SE in the setting of pneumothorax is usually related to ongoing air leakage, insufficient pleural decompression, or drainage‐related factors, such as tube malposition, inadequate observation after clamping, or early removal [21]. In our patient, the drainage site may have been vulnerable to postoperative air leakage because of the marked paucity of subcutaneous tissue, incomplete closure of the deeper soft tissues, and the relatively large subxiphoid incision. Due to the fact that the right mediastinal pleura had also been entered during surgery, leakage through the wound was considered one possible explanation for the second episode of pneumothorax, although a definitive causal relationship could not be established. Small‐bore pigtail catheters may offer various advantages, such as the creation of a smaller wound, less insertion discomfort, and potentially less wound‐related air leakage. However, their role in patients with rapid air leakage or suspected bullous rupture remains debated [17, 22]. Due to the fact that their narrow lumen may compromise evacuation efficiency and increase the risk of blockage, they may be less suitable in selected patients with substantial pleural air leakage. In such situations, inadequate drainage may facilitate the recurrence of pneumothorax and worsening SE. These considerations highlight the importance of tailoring drainage management to the clinical scenario rather than relying on a single approach for all patients.

SE is often self‐limited, and no standardized treatment strategy has been established. Management should be individualized based on symptom severity, the extent of SE, the source and likelihood of the ongoing air leakage, and the effectiveness of pleural decompression, with the primary goals of treatment involving symptom relief, adequate drainage, and control of continued air escape [5, 23]. Although extensive SE may cause substantial discomfort and prolonged hospitalization, major complications are uncommon [6, 24]. In severe or progressive cases, therapeutic options include optimization of pleural drainage, application of suction, subcutaneous drain placement, or decompressive skin incisions [25, 26, 27]. The choice of intervention should be individualized according to the patient's symptoms and the presumed mechanism of disease. In our patient, the cervical symptoms promptly improved after chest drainage; moreover, the pleural effusion volume was limited, and the abdominal SE was not considered to be life‐threatening. Therefore, additional subcutaneous drainage was not performed. However, the favorable clinical course observed in this case does not establish the superiority or general applicability of conservative treatment. Moreover, due to the fact that this patient required emergency surgery for acute type A AoD, the recurrent pneumothorax should not be interpreted simply as a consequence of perioperative management alone.

## Author Contributions

**Qiulin Ran:** formal analysis, investigation, visualization, writing – original draft. **Zanxin Wang:** methodology, supervision. **Minxin Wei:** methodology, supervision. **Bin You:** conceptualization, formal analysis, supervision, validation, writing – review and editing.

## Funding

The authors have nothing to report.

## Disclosure

We declare that none of the authors listed on the manuscript are employed by a government agency that has a primary function other than research and/or education. None of the authors submitting this manuscript are acting as an official representative or on behalf of the government.

## Consent

Written informed consent for patient information and images to be published was provided by the patient.

## Conflicts of Interest

The authors declare no conflicts of interest.

## Data Availability

The data presented in this study are available on request from the corresponding author.
